# Informing Dementia Support Programs That Serve Low-Income, Multilingual Communities in a Safety Net Health System: Use of Focus Groups to Identify Specific Needs

**DOI:** 10.3390/geriatrics9020033

**Published:** 2024-03-06

**Authors:** Andrew Pak, Abriella Demanes, Shirley Wu, Katherine Ward, Mailee Hess

**Affiliations:** 1 Department of Medicine, Harbor-UCLA Medical Center, Torrance, CA 90502, USA; 2 Section of Geriatrics, Division of General Internal Medicine, Department of Medicine, Harbor-UCLA Medical Center, Torrance, CA 90502, USA; 3 Department of Medicine, UCLA David Geffen School of Medicine, Los Angeles, CA 90024, USA; 4 Geriatrics Section in the Department of Medicine, Division of Primary Care and Population Health, Stanford University School of Medicine, Stanford, CA 94305, USA

**Keywords:** dementia, caregiver, dementia support program, safety net, unmet needs, barriers, multicultural, Spanish-speaking

## Abstract

The Centers of Medicare and Medicaid Services recently announced a new voluntary nationwide model. This model aims to provide comprehensive, standard care for people living with dementia and their unpaid caregivers and to enhance health equity in dementia care. However, little is known about the needs of older adults with dementia and their caregivers in a multiethnic and multicultural patient population of a safety net health system. The aim of this study is to include their voices. We conducted four focus groups in English and Spanish to investigate the common needs and barriers unique to the care of patients within the Los Angeles County healthcare system. Using qualitative, iterative analyses of the transcripts, we identified four domains of concern from the dyads (persons with dementia and their caregivers): need for education for dyad-centered care, barriers to resources, dyad safety, and caregiver burden and insight. These domains are interconnected, and the way this patient population experiences these domains may differ compared to those in well-resourced or predominantly English-speaking healthcare settings. Therefore, the identified domains serve as potential building blocks for dementia support programs inclusive of underserved, multicultural populations.

## 1. Introduction

As of 2023, about 6.7 million Americans are living with dementia, which is projected to nearly double to 13 million people by 2050. In the United States, the Hispanic older adult population has been projected to grow the fastest and has a significantly higher rate of dementia compared to White older adults [1]. In Los Angeles County (LAC), the most populous and diverse county in the United States, there are nearly 200,000 residents living with dementia [2]. As the second largest municipal healthcare system in the nation, the LAC Department of Health Services (DHS) provides care for low-income patients on Medicaid (Medi-Cal) and those without insurance [3].

In the United States, nearly half of all self-identified caregivers who provide help to older adults do so for those with dementia [1]. Most caregivers are family members and are often considered the invisible second patients, coupled with the person with dementia (PWD) as a dyad [4]. Previous studies have identified dyads’ general needs and barriers to care, which include lack of family support, financial constraints, accessibility to support services, caregiver burnout, difficulty with psychosomatic symptoms of dementia, and lack of knowledge about dementia [5,6,7,8]. Prior studies have also depicted experiences of dyads from various ethnic backgrounds. In particular, when characterizing dyads from Hispanic families, language barriers, cultural beliefs towards dementia, and family values have been shown to (a) influence the understanding of dementia as a disease, (b) delay the diagnosis of dementia, (c) lead to underreporting of dementia-related behaviors and underutilization of resources, and (d) increase the burden of care [9,10,11,12,13,14,15,16]. However, little is known about the experiences of vulnerable, multicultural dyads in a safety net health system regarding their dementia care and the quality of support, if any, that they receive. We aim to explore their current state of need and experience.

Several dementia support group programs have previously been established. Recognizing the importance of these programs, the Centers of Medicare and Medicaid Services announced in July of 2023 a new voluntary nationwide model called Guiding an Improved Dementia Experience (GUIDE) [17]. GUIDE aims to provide a comprehensive, standard approach to care for people living with dementia and their unpaid caregivers and enhance health equity in dementia care. However, dyads in a multiethnic and multicultural patient population have been significantly underrepresented in previous studies of dementia support programs [18]. This deficiency is even more pronounced in the context of a safety net health setting.

Through focus group-based qualitative analyses, we build upon the established needs and challenges dyads have conventionally faced to elicit common and unique barriers to care within the LAC-DHS patient population. By better understanding the barriers these patients face, we aim to implement interventions to better support the dyads.

## 2. Materials and Methods

### 2.1. Study Design, Setting and Participants

The research team created a focus group guide that started with open-ended questions regarding cultural beliefs around dementia, barriers dyads experience in accessing care and resources, and what kind of support dyads currently have (Table 1). Subsequently, more targeted questions were asked regarding specific needs and resources. 

Participants were recruited from LA County geriatrics clinics. Patients and caregivers were eligible to participate if the patient had a documented diagnosis of dementia, lived in the community, and the patient and caregiver spoke English or Spanish. Participants with dementia and their caregivers were able to give informed consent. Caregiver participants could be unpaid family members or friends, or paid caregivers, and could care for a PWD at any stage of the disease.

Focus group discussions were led by a licensed social worker who was fluent in both English and Spanish.

### 2.2. Data Collection and Analysis

Four focus group discussions were held in February 2021 with a total of 11 participants. The groups were divided based on the participants’ preferred availability. 

Due to the SARS-CoV-2 pandemic, all discussions were conducted and recorded through a video communication platform. The videos of all eligible participants were turned off, and no legal names were represented in the final transcription. The recordings were then transcribed into English.

Using qualitative analysis software, ATLAS.ti (version 9, Scientific Software Development GmbH, Berlin, Germany), the transcripts were independently coded line-by-line by three study investigators using both deductive and inductive coding approaches. An initial set of codes was defined by the research team and refined after iterative analyses of the transcripts. Based on the coding representing the dyad’s experience and needs, four major domains were identified and discussed in this article. Emerging themes and exemplary texts were discussed among the full study team and any differences in coding were settled by group consensus.

## 3. Results

### 3.1. Participant Characteristics

Of the ten caregivers and one PWD, nine were primarily Spanish-speaking. Among the caregivers, two were wives of PWDs, six were daughters, and one caregiver was a son. The one PWD who participated was in an early stage of dementia. Nine caregivers cared for PWDs in the moderate to late stages of dementia, while only one caregiver cared for a PWD in an early stage. 

### 3.2. Domains 

Based on iterative analyses of the transcripts, four major domains based on dyad’s experiences and needs were identified: (1) the need for education about dementia, (2) barriers to accessing resources, (3) dyad safety, and (4) caregiver burden and insight. The topics discussed and highlighted under each domain is summarized in Table 2.

#### 3.2.1. Need for Education about Dementia 

Addressing deficiency in knowledge about dementia was identified as a major need for dyads. Dyads are often confronted with cultural beliefs around dementia that are inaccurate and build stigma and shame around the disease. A PWD shared her own understanding of dementia as follows:

“Dementia to me was like—I figured it was a bit like someone being crazy.”

When these negative views of the illness came from family members and relatives of the PWD, participants shared that this led to them tending to hide the illness.

“And I think it has to do with the fact that there is so much taboo around [dementia]… and that when people were to get dementia in the old days nobody spoke about it. It was kind of just hush hush, and it just wasn’t spoken about. It was just something that was just shoved under the rug and that’s it.”

Dyads expressed a desire to learn more about dementia, not just to address inaccurate cultural beliefs but to better care for their loved one with dementia. However, they felt that the educational component within the care they received was insufficient. Participants shared that even though primary care doctors of the PWD or caregiver often offer sympathy and brief statements of emotional support during the visit, the support does not extend beyond that.

“Yeah, [the doctors] don’t take [caring for a PWD] into account—they just told me that they are sorry because it is very serious and that they know that it is something very sad for us… they just said ‘I’m sorry’ because it takes a lot to take care of someone with this.”

Even when a caregiver receives information on dementia, the participants have shared that the information is often insufficient and not specific enough to care for a PWD. For instance, when asked who provided information about dementia, a caretaker responded:

“Well, the doctor… when [the PWD] had [dementia] they explained a little bit… but up until now we haven’t received anything. I would like to know more—to know more to be able to take care of him and orient him, like how one can have patience to take care of them.”

#### 3.2.2. Barriers to Accessing Care and Resources

Three major barriers were identified in accessing resources: (1) money, (2) language, and (3) insurance.

Nearly all caregivers mentioned personal finances as a barrier to resources for the PWD. The dyads shared a wide array of aspects of care impacted by financial limitations, including small living spaces, delays in installation of safety equipment like railings and shower bars, inability to pay for caregiver services, or limited transportation for medical appointments. All dyads denied facing food insecurity. When a wife of a PWD was asked about the biggest barriers to receiving the proper healthcare for her husband, she simply stated:

“Well, … the money… unfortunately, we don’t make enough money where we can pay someone privately that can come take care of [my husband].”

Access to resources was often limited due to information not being available in Spanish. When asked about receiving social support for the PWD, a caregiver shared:

“Well, honestly, I didn’t really know about much support in the beginning, and when I did try to get help, they didn’t have any in Spanish—and in this house we only speak Spanish. So then anywhere that [the PWD] could go would be in English in majority, so I didn’t receive any help before.”

Even when care is provided with the help of a language interpreter, the dyads shared a level of disparity in either the quality of communication or degree of advocacy for the PWD. The dyads noted that interpreter services used to be in-person; however, due to the SARS-CoV-2 pandemic, they were offered to them remotely via phone, which the participants felt was inferior to in-person interpretation. When a caregiver was asked if the interpreter service was over the phone or in-person, she responded:

“No… no… [the interpreter service] is over the phone… before it was in person, which I feel is way better than over the phone. Over the phone… my mother can’t really hear, and when they ask her things… she struggles. As opposed to when it was an in-person translator it was much better.”

Dyads even expressed a mistrust of the medical community when dyads were primarily Spanish speaking. A daughter of a PWD further elaborated her concerns regarding advocating for her father’s access to information and resources when accompanied by her mother alone, who only speaks Spanish.

“I like to make sure that I am there for every single appointment, and that I talk to with whoever I need to speak to and that if there is anything [the PWD] needs, he’s getting it, and I’m advocating for it, because my mom speaks Spanish, and she looks older. You know, people take advantage of her. So then I just, I’m there to make sure that doesn’t happen.”

Though not applicable to all PWD in the focus group, immigration status was identified as an important barrier to care. At least one PWD did not have access to Medi-Cal and covered programs for five years given the federal law requiring a minimum of five-years of continuous residency prior to enrollment. The caregiver shared that her mother did not qualify for various resources recommended by the social worker due to her immigration status.

“In my mother’s case, last year, she still did not qualify because she had to have a minimum of 5 years of residency and that’s why she didn’t qualify. But this year, it’s been 5 years so she qualifies now. That is another reason why I couldn’t do it before. So that is a huge barrier for people that have that issue and they really do not receive any help from the government for the very reason of their immigration status.”

#### 3.2.3. Dyad Safety 

Physical safety concerns were a recurrent theme for both the PWD and the caregiver. These concerns impacted the dyad’s ability to attend medical appointments and to follow through on treatment plans. 

Falls and wandering were major issues for the physical safety of the PWD. While poor vision and lack of hearing aids were identified as contributing to fall risk, there were issues related to the medical center, like parking problems, that impacted a dyad’s ability to safely come to medical appointments. Five caregivers reported concerns around high fall risk situations on the medical center campus, such as having to walk down six flights of stairs in a parking lot to attend a doctor’s appointment. Caregivers shared fears of the PWD wandering away or crossing the street unsafely if they were to drop off the PWD while they look for parking. 

Safety concerns were not just for the PWD. There was significant discussion around the safety of the caregiver as well. This often pertained to behavioral issues of the PWD not adequately being considered or addressed by providers. 

For example, a daughter of a PWD who has insulin-resistant diabetes mentioned that “food is a big trigger” for his verbal and physical aggression:

“Prior to his stroke, he was pretty much in charge. And now he has his wife and his youngest daughter telling him what he can and cannot eat. And in his, you know, “machista” [male chauvinist] way, this is the worst thing that could possibly have happened to him.”

As a way of mitigating risks of injury for the dyad, the daughter shared that she taught her mother the following evasion strategy:

“We’ve told her to run. We’ve told her that if it’s food that he wants, to just give it to him, you know. We rather his sugar be 300 than, you know, [her mother] be hurt or [her father] fall or something like that. You know, just avoid the worst possible scenario.”

Similar to how food restriction of this PWD with diabetes created an unintended secondary risk, a daughter of another PWD shared that even an assistive device like her father’s cane can be dangerous. Before being told by the daughter, the PWD’s physical therapist was unaware of such risk:

“Actually, every time [my father] has therapy, and a therapist comes in here and asks why isn’t he using a cane? Umm… I let them know like it’s a weapon. It’s not medical equipment if it can be a weapon.”

The caregivers did not receive specific instructions from providers on how to mitigate these behavioral symptoms that put the dyads at potential physical harm. One caregiver had an occupational background in de-escalation techniques, but the majority did not have such training. 

#### 3.2.4. Caregiver Burden and Insight 

Examples of caregiver burden shared by the participants included the need for continuous supervision of the PWD, multiple doctor’s visits while balancing their own work and family, navigation of the healthcare system, barriers to resources, the physical and mental toll of caregiving, and limited family support. They also expressed a cultural preference for family-based care and had negative views of institutional care, which places even more weight and responsibility on the caregiver. 

All family members of PWDs described receiving some assistance from other family members by either splitting the time of caregiving or sharing the role; however, they felt that the help was not enough to offset the burden of primary caregiving. Two of the caregivers sought the services of In-Home Supportive Services (IHSS), but both shared a negative experience. One of the caregivers, who had interviewed about fifteen people, stated the following:

“I ultimately decided [to hire a person] because I figured my father would benefit from his companionship. But I did need him to do things like administer medicine, measure his blood sugar, measure his blood pressure, administer his insulin. And when the day came, he wasn’t able to do that… He was opposed to my father drinking medicine, which I was really upset at because the last thing we need is someone to tell my dad not to take medicine.”

Despite the challenges, the caregivers shared important insights into caregiving for PWDs, recognizing that the care is individualized and deeply personal:

“[There is a sense of] responsibility, love, [and] care… because it’s someone who has a lot of value, and so we need to really take care of them even if others don’t do it.”

A daughter of a PWD shared that she does not see herself as a caregiver, nor does she see her mother as a PWD:

“I just see it like her just being my mom.”

In terms of the individualization of care, a caregiver shared that understanding how each dyad’s circumstance is unique helped him better personalize the information perceived.

“From what I’ve seen, I’ve read all of this stuff online and half of the stuff applied to us and the other half of stuff doesn’t apply to us. So it gives me the idea that everybody encounters different things with dementia, and whether it’s your parents, grandparents, your wife, your husband, their needs will be different than someone else’s needs. They may have the same dementia but they may handle it differently… the family handles it differently, the patient handles it differently, I don’t think any two patients are probably alike.”

Furthermore, a caregiver acknowledged that aging is a shared experience not only among PWD but also among the caregivers and that she would like to learn more about dementia and aging. She aspired to educate her children as well and to show “what it would be like if one lived longer than others”. She concluded by endorsing that the longevity of a healthy mind should not be taken for granted.

“There are people that live into their 80s, 90s, 95, more than 100, and they have their mind okay and are able to focus. But some of us don’t have that privilege. The privilege to have their mind privileged.”

## 4. Discussion

Using focus groups, we identified four domains (the need for education about dementia, barriers to accessing resources, dyad safety, and caregiver burden and insight) that contribute to dyad difficulty with accessing care and resources. We recognize that the domains are not separate but rather interconnected. Additionally, these domains align well with the general categories of challenges that have been previously studied with other dyad populations [5,6,7,8]. What separates these domains are the notable topics discussed within each domain, as summarized in Table 2, and how they represent the specific needs of dyads in a safety net health system such as LAC-DHS.

One of the best interventions to date for addressing the needs of dyads are dementia support programs. Several dementia support programs have been established and show positive outcomes for both PWDs and caregivers, including decreased caregiver depression, improved quality of life for PWDs, and decreased healthcare utilization [19,20,21,22,23,24,25]. However, only two studies specifically targeted minority populations using an online or videophone platform. Neither of these studies investigated the specific needs of the dyad population prior to implementing a technology-based support system or included participants specifically from a safety net health system. Further work should be done on dementia support programs in safety net populations, as these programs offer the potential to address almost all the identified needs in this study.

Providing dyads with education about dementia and dementia care is clearly an important aspect of dementia care, no matter which healthcare settings. However, for Spanish-speaking populations served by LAC-DHS, part of this education may also require directly addressing cultural beliefs around dementia and equipping caregivers with tools to educate other family members. Our focus groups identified that many of the historically negative views of dementia, and equating a dementia diagnosis with being “crazy”, can limit family support and may even limit a family’s acceptance of the diagnosis [9,10,11,12,26]. As this cultural view of dementia is a fundamental step in accepting dementia as a pathologic disease, we recommend early exploration and education on cultural beliefs of dementia for Hispanic dyads at the time of diagnosis. Furthermore, many providers at LAC-DHS do not share the same cultural background as the dyads. A successful dementia support program would help bridge this gap by not only educating dyads about dementia and breaking down cultural beliefs around the illness, but also helping to educate providers about these cultural beliefs. Utilizing community health workers as part of the dementia support program team may help facilitate this [27,28,29]. Future studies should explore whether the effects of addressing cultural beliefs around dementia early through a dementia support program improve access to care or caregiver burden.

We also identified that further education for providers is also necessary and critical. While providers offer empathy for the diagnosis, they are not providing meaningful education about the diagnosis or management of the disease, especially when addressing the behavioral symptoms of PWDs. This aligns with a 2020 report from the Alzheimer’s Association which revealed that 50% of surveyed primary care providers expressed being uncomfortable with the management of dementia [1]. As the behavioral symptoms of a PWD also affect the physical safety of both the caregiver and the PWD, we recognize a particular need for improvement in educating providers on situational harm reduction. 

Situational harm reduction requires that medical professionals use clinical discretion and a holistic view when making medical management recommendations and integrate de-escalation techniques. For example, in the case of the PWD with diabetes mellitus, diet restriction, frequent blood glucose checks, and insulin injections intensified behavioral symptoms that led to aggression toward his caregiver. This was exacerbated by a shift in family dynamic when the formerly machista (male chauvinist) PWD is told what to do by his wife and daughters. Transitioning from guideline-directed medical management, such as strict hemoglobin A1c goals, to focusing on harm reduction and recognizing cultural family dynamics may help to facilitate more beneficial care. Similarly, if a walking-assistive device serves as a weapon that can potentially harm the caregiver, a therapist could explore alternative mobility aids, such as walking behind a wheelchair, which would be more difficult to pick up and use as a weapon. The more comfortable providers are with managing dementia, the more helpful their recommendations may be in managing comorbid chronic conditions. The need for emphasis on situational harm reduction and education of providers is a recommendation for dementia care in all healthcare settings. However, dementia support programs can assist with this issue by advocating for dyads when communicating with providers, helping to call attention to safety issues.

Overall, challenges like language barriers affecting education and access to resources, cultural views on dementia influencing the understanding of dementia as a disease, and preference for family care over institutional services were consistent with previous studies that examined dyads from Hispanic families [11,12,16,30]. With such a high percentage of dyads whose first language is not English, language was perhaps not a surprising barrier. Interestingly, however, language continues to be a significant factor despite access to phone interpreters in LAC-DHS. Even though in-person interpreters are becoming more available at LAC-DHS since the SARS-CoV-2 pandemic, most patient interactions still occur through phone interpreters due to relatively limited availability of in-person interpreters. Dyads in this study voiced a strong preference for in-person interpreters, which likely would facilitate more effective and efficient physician encounters and communication. When a language congruent provider or team member is not available, we recommend the use of an in-person interpreter. This can help eliminate barriers to communication inherent to phone interpreters, including PWDs not understanding how to use the phone, limited volume making it difficult for those with hearing impairment to hear, and the inability to pick up nuances of body language and facial expressions [31]. Improving the quality of interpretation services may also help mitigate the mistrust in the medical community that younger caregivers shared with regard to their elderly parents potentially being taken advantage of because they are less able to speak English.

Immigration status and its impact on insurance has significant implications for the population served by LAC-DHS. As a safety net hospital system, LAC provides care for patients even if they do not have insurance. However, there are many resources that California’s Medicaid program covers that are not available to uninsured patients, in particular the In-Home Supportive Services (IHSS) program, which provides money for caregiving services and for many families is a source of income for the primary caregiver. Financial strain was consistently highlighted as a significant burden for the families participating in these focus groups. Access to programs such as IHSS play a critical role in addressing this pressing need. Since the completion of this study in February 2021, a new law in California came into effect in May 2022 that allowed adults 50 years and older to receive full-scope Medi-Cal regardless of immigration status. Future research should explore whether this law has had a significant impact on dyads accessing needed resources for dementia care [32]. 

A notable dementia support program model for adaptation for dyads in a safety net health setting is the Care Ecosystem program developed by the University of California, San Francisco. It provides education for dyads, screenings for common problems like behavioral issues, and education on how to manage these issues, as well as connection to resources and care coordination [22]. However, for a dementia support program like this to succeed in a safety net setting, it would need to (1) incorporate resources that cater to non-English speaking dyads with cultural competency, (2) emphasize harm reduction strategies when dyads report that providers are not recognizing the safety concerns of the dyads, (3) utilize language-congruent staff and help advocate for in-person interpreters during clinic visits, and (4) maximize financial resources for dyads including IHSS and other unique programs of Medicaid.

## 5. Conclusions

The voices of multiethnic and multicultural dyads in a safety net health system have been significantly underrepresented previously. Through our study, we hope to include their voices and highlight their specific needs. We recommend utilization of established dementia support program models and molding them to fit the specific needs of the safety net patient population. By providing high-quality, intentional care through a dementia support program for vulnerable dyads, we hope to reduce health disparities and promote equal access to resources. Future study is needed to characterize the effects of the implementation of such dementia support programs in safety net health settings. In the future, it will also be imperative to study how the nationwide GUIDE model is meeting the needs of low-income, multicultural populations. At this time, GUIDE participants are limited to Medicare Part B providers, and the model excludes Medicaid-only patients served by the LAC-DHS. We hope that this study inspires and serves as a building block to provide equitable dementia care to underrepresented dyads across the United States.

## 6. Limitations

The limitations of this study include the small sample size of the focus group, with most of the PWDs in moderate to late stages of dementia. Though sampling of earlier stages of dementia was limited, the needs of PWDs with advanced disease place a heavier burden on the caregiver and require most assistance in education and coordination of care. Given limited research in this area, especially for safety net patient populations, identification of the needs of those with advanced disease was prioritized. 

This study also welcomed participants whose first language is not Spanish and may not identify culturally and ethnically as Hispanic. Though the majority of participants spoke Spanish or both Spanish and English, we recognize that the cultural view of dementia discussed by this cohort of participants cannot be generalized across all patient population of LAC-DHS or other safety net health systems. This study also included both unpaid and paid caregivers, who may have provided an opportunity to explore different perspectives and incentives. In our review of the transcripts, the one paid caregiver did not contribute significantly to the focus group discussion, and no direct statement was used in our results.

Another limitation arises from the fact that the focus group interviews were conducted during the SARS-CoV-2 pandemic, which required all focus groups to be conducted virtually through a video communication platform. However, the videos of all participants were turned off for personal information confidentiality. At times, the transcripts reflect moments of silence and reduced participation. Given the lack of visualization of body language and facial expressions, which contribute greatly to the depth of conversations, the facilitation of the natural flow of conversation was not optimized, and some valuable information or insight might have been withheld. Additionally, this limited the participation of PWDs who did not understand how to use the virtual technology. 

The SARS-CoV-2 pandemic also impacted the resources for dyads: dyads described worsened financial limitations and lack of in-person interpreters during provider visits, and resources such as adult day centers were not open during the pandemic, which limited daytime respite care for PWDs. There have been studies looking at the negative effects the pandemic had on dementia caregiving and the adaptations made to mitigate the impacts [33]. We acknowledge that the restoration of the availability and accessibility of healthcare resources since the discontinuation of mandated isolations for SARS-CoV-2 may influence the applicability of some of the needs identified in this study. The domains of the needs of dyads characterized in this study, however, apply to dyad-centered care regardless of the status of the global pandemic.

## Figures and Tables

**Table 1:** Example guide questions asked in English or Spanish to participating dyads during focus groups.

Anticipated Discussion Topics	Example Guide Questions
Understanding of dementia	- How is dementia viewed in your culture? - When you hear the word “dementia”, what comes to your mind?
Barriers to care	- What are your biggest barriers as a family? - Do you have issues gaining access to proper nutrition? - Do you experience financial issues in providing anything necessary for the home? - How do you get to the doctor?
Support system	- What kind of social supports do you have? - Have you talked to your personal doctor about being a caregiver for someone with dementia? - Have social workers connected you with resources to help the PWD? - If you had a magic wand that would give you the assistance that you needed the most, what would that magic wand give you?

**Table 2:** Summary of key discussions under each domain that highlights experiences most pertinent to the dyad participants receiving dementia care at LAC-DHS.

Domains	Key Topics Discussed
Need for education about dementia	Cultural taboo around dementia Insufficient practical information about caring for a PWD from primary care physicians
Barriers to accessing resources	Financial limitation at forefront Resources unavailable in Spanish Phone Spanish interpretation not as effective as in-person interpreters Mistrust in medical community of younger caregivers revolving around PWD’s inability to speak English at doctor’s visits Influence of immigration status on resource qualification
Dyad safety	Challenges with parking posing fall and wandering risks for PWDs Caregiver safety concern from PWDs’ behavioral symptoms Behavioral symptoms exacerbated by a change in cultural family dynamic Safety equipment being used a weapon against caregivers
Caregiver burden and insight	Factors contributing to caregiver burden: ○ Caregiver’s need for constant supervision of PWD ○ Navigating health systems and multiple doctor’s visits while balancing caregiver’s own work and family ○ Limited family support ○ Cultural preference for family-based care; negative views on institutional care ○ Negative experiences with hiring caregivers through In-Home Supportive Services Shared insight as caregivers: ○ Dementia care should be individualized ○ Privilege of having a healthy mind

## Data Availability

The transcripts were coded using ATLAS.ti, a proprietary, computer-assisted qualitative data analysis software. The coding data can be available upon request to corresponding author.
